# Influence of Li_2_O Incrementation on Mechanical and Gamma-Ray Shielding Characteristics of a TeO_2_-As_2_O_3_-B_2_O_3_ Glass System

**DOI:** 10.3390/ma14144060

**Published:** 2021-07-20

**Authors:** Aljawhara H. Almuqrin, Mohamed Y. Hanfi, M. I. Sayyed, K. G. Mahmoud, Hanan Al-Ghamdi, Dalal Abdullah Aloraini

**Affiliations:** 1 Department of Physics, College of Science, Princess Nourah bint Abdulrahman University, Riyadh 11671, Saudi Arabia; ahalmoqren@pnu.edu.sa (A.H.A.); hmalghmdi@pnu.edu.sa (H.A.-G.); daalorainy@pnu.edu.sa (D.A.A.); 2 Institute of Physics and Technology, Ural Federal University, St. Mira, 19, 620002 Yekaterinburg, Russia; mokhamed.khanfi@urfu.ru; 3 Department of Nuclear Medicine Research, Institute for Research and Medical Consultations, Imam Abdulrahman bin Faisal University, Dammam 31441, Saudi Arabia; 4 Department of Nuclear Power Plants and Renewable Energy Sources, Ural Power Engineering Institute, Ural Federal University, St. Mira 19, 620002 Yekaterinburg, Russia; kmakhmud@urfu.ru

**Keywords:** mechanical properties, shielding properties, elastic moduli, Monte Carlo simulation

## Abstract

According to the Makishema–Mackenzie model assumption, the dissociation energy and packing density for a quaternary TeO_2_-As_2_O_3_-B_2_O_3_-Li_2_O glass system were evaluated. The dissociation energy rose from 67.07 to 71.85 kJ/cm^3^, whereas the packing factor decreased from 16.55 to 15.21 cm^3^/mol associated with the replacement of TeO_2_ by LiO_2_ compounds. Thus, as a result, the elastic moduli (longitudinal, shear, Young, and bulk) were enhanced by increasing the LiO_2_ insertion. Based on the estimated elastic moduli, mechanical properties such as the Poisson ratio, microhardness, longitudinal velocity, shear velocity, and softening temperature were evaluated for the investigated glass samples. In order to evaluate the studied glasses’ gamma-ray shield capacity, the MCNP-5 code, as well as a theoretical Phy-X/PSD program, were applied. The best shielding capacity was achieved for the glass system containing 25 mol% of TeO_2,_ while the lowest ability was obtained for the glass sample with a TeO_2_ concentration of 5 mol%. Furthermore, a correlation between the studied glasses’ microhardness and linear attenuation coefficient was performed versus the LiO_2_ concentration to select the glass sample which possesses a suitable mechanical and shielding capacity.

## 1. Introduction

The field of radiation physics concerns the development of protective materials that are used to absorb radiation. These radiation shields are defined as any material used to attenuate photons, and are typically placed between the radiation source and the worker or patient. These shields are becoming increasingly more necessary as more fields begin using radiation on a daily basis [1,2,3,4,5]. Fields such as medicine, food conservation, and agriculture all rely upon radiation to fully function. Despite the benefits of radiation present across various fields of work, high-energy particles or ionizing radiation can be extremely harmful to the human body if underexposed for a long time. Some side effects of radiation exposure may include acute radiation syndrome, cutaneous radiation injuries, and cancer development. Radiation shields work to minimize these effects and protect humans that may come into contact with ionizing radiation [6,7,8,9].

When selecting a radiation shield for a specific application, several characteristics of how the radiation is being used must be known to utilize the best possible material. For example, concrete is commonly used against X-rays and neutrons, and is ideal for lining the walls of rooms. These properties make concretes suitable for lining the walls of nuclear reactors, for instance, but their lack of mobility and tendency to crack makes them unideal for other applications [10,11]. Other materials that have been used as radiation shields include alloys, composites, construction materials (such as granite and marble), and glass [12,13,14,15,16].

Glasses offer advantageous properties over other radiation shields because, in addition to their excellent shielding ability, they are transparent, are simple to manufacture, and can have a wide range of possible compositions. The structural, chemical, and shielding features of glasses can be changed by doping metal oxides and heavy metal oxides (HMOs) into the glass system. Metal oxides and HMOs act differently on the glass matrix, depending on their characteristics. There exist three different kinds of oxides: network formers, network modifiers, and intermediates [17,18,19].

Borate is commonly used as a glass former and is found in most commercial glasses. Borate can easily modify its coordination with three of four oxygen units to provide a strong and adjustable structural composition. This change increases its chemical and thermal resistance by creating non-bridging oxygens (NBOs). Additionally, borate glasses have a low viscosity, high chemical durability, high visible light transparency, low cost, and good mechanical stability. Despite the abilities of borate, it has a low density, which is undesirable by itself. Other metal oxides and HMOs are added to borate glasses to improve their density [20,21].

Tellurite, or TeO_2_, is a glass intermediate with a high dielectric constant, a good electrical conductivity, transparency in visible and infrared light, and good solubility of rare-earth ions. Tellurite glasses can be used as planar waveguides and optical amplifiers in optical applications, but have also been used in radiation shielding applications. Tellurite has a high density and atomic number, making it a HMO [22,23]. Due to tellurite being an intermediate, it does not form a stable glass system by itself due to its weak connectivity. By pairing TeO_2_ with oxides, such as B_2_O_3_, its stability can be increased. Borotellurite glasses have high stability and transparency and are used in fiber-optic communication systems and nonlinear optical devices.

In order to assess the capability of a medium to become a radiation shield, several parameters must be calculated and analyzed. These parameters are typically calculated experimentally, but simulations are often used to validate the obtained data. Simulations also offer the ability to test samples before spending the time and cost fabricating them, and if they demonstrate potential, they can then be experimentally examined. Simulations have also proven to be extremely reliable in correctly assessing the shielding ability of a sample across a wide range of energies [24,25].

The novelty of the present study lies in the application of the Makishima–Mackenzie (M–M) model to predict the elastic and mechanical characteristics of As_2_O_3_-B_2_O_3_-Li_2_O-based tellurite glasses. Moreover, the radiation shielding properties of the mentioned glasses were evaluated using the Monte Carlo simulation method. Additionally, the accumulation of photons in air and inside the investigated glasses was calculated via the Phy-X/PSD program.

## 2. Materials and Methods

### 2.1. Mechanical Properties

The mechanical features (Poisson ratio, microhardness, fractal bond conductivity, and longitudinal and shear velocities), as well as elastic moduli (EM) (Young, shear, longitudinal, and bulk), were investigated for five glass samples reported by [26]. The mentioned glass system contained TeO_2_-As_2_O_3_-B_2_O_3_-Li_2_O in composition. Based on the foundations approved by Makishema and Mackenzie [27,28], the EM were computed. The dissociation energy (G_t_) is a measure for the heat of formations (enthalpy) required to fabricated the glass system. The following equation describes it.

(1) $$\mathrm{Gt}\;\left(\frac{\mathrm{kJ}}{{\mathrm{cm}}^{3}}\right)=\sum^{\;}X_{i}G_{i}$$

X_i_ is fractional by mol of the constating compounds. The packing density is an essential factor related to the oxide and metal ionic radius R_o_ and R_M_. The V_t_ is evaluated using Equation (2), where V_i_ is the packing factor of the constituting compounds.

(2) $$\mathrm{Vt}=\left(\frac{\rho }{\mathrm{MW}}\right)\times \sum^{\;}X_{i}V_{i}$$

The previously calculated values for G_t_ and V_t_ were used to compute Young (E), shear (K), longitudinal (L), and bulk (B) modules, as presented in Equations (3)–(6). In addition, some mechanical properties based on that derived from the EM were evaluated in Equations (7)–(9), such as the Poisson ratio (σ), the microhardness (H), softening temperature (T_s_), and fractal bond connectivity (d) [29].

(3) $$E=2{\;V}_{t}G\;$$

(4) $$B=1.2{\;V}_{t}E\;$$

(5) $$S=\frac{3\;\mathrm{EB}}{\left(9B-E\right)}\;$$

(6) $$L=B+\frac{3}{4}\;S\;$$

(7) $$\sigma =0.5-\frac{1}{7.2}V_{t}\;$$

(8) $$H=\frac{\left(1-2\sigma \right)}{6\left(1+\sigma \right)}\;$$

(9) $$T_{s}=\frac{M_{W}}{\left(\rho_{\mathrm{glass}}\times \;C\;\right)}\times V_{s}^{2}\;$$

### 2.2. Gamma-Ray Simulation and Theoretical Calculations

**Figure 1:**
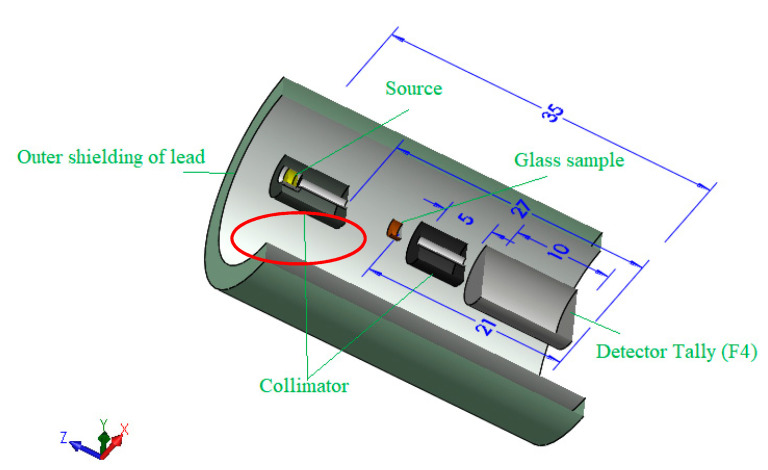
The Monte Carlo MCNP 3D simulation geometry is used in the present work.

The present study’s second aim is to report the radiation protection capacity for the investigated TABLi samples. In order to achieve the desired target, the MCNP-5 [30] and a theoretical calculation program named Phy-X/PSD [31] were used to evaluate the protection ability. Both previous programs used the chemical compositions and densities of the investigated glasses to evaluate the shielding factors. On the other hand, there are differences in the nuclear libraries, which used to extract the interaction cross-sections. The MCNP-5 used ENDF/B-VI.8 as a primary source, but the Phy-X/PSD used only the NIST database. The geometry used in the MCNP-5 simulation was illustrated in Figure 1 and discussed in detail in many publications [32,33,34]. Additionally, the investigated glasses’ chemical compositions were given in Table 1.

## 3. Results and Discussion 

### 3.1. Mechanical Properties

**Figure 2:**
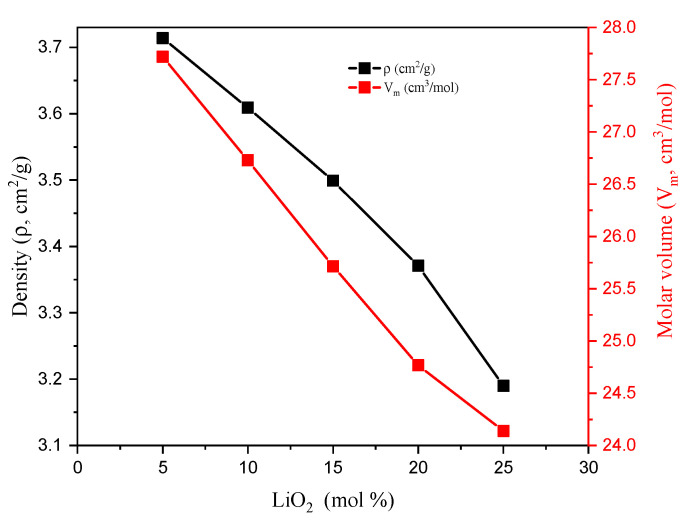
Variation in the density and molar volume versus the LiO_2_ concentration.

The selected TABLi samples have a density (ρ, g/cm^3^) that decreased linearly from 3.714 to 3.190 g/cm^3^, as shown in Figure 2. The decrease in the glass density is related to the compactness of the glass materials, which is predicted through the distribution density for boron, D (B), and distribution density for Li, D (Li). The D (B) and D (Li) were calculated and showed in Table 2, where both D (B) and D (Li) increased with an increasing Li_2_O concentration in the glass network. Thus, the density of the samples decreased. Table 2 also showed that the boron-boron separation, r (B-B), lithium-lithium separation, r (Li-Li), and tellurium-tellurium separation, r (Te-Te), decreased with an increasing Li_2_O concentration in the glass network. This can be ascribed to the replacement of Te ions with a higher ionic radius (R_Te_ = 2.22 Å) by a smaller Pauli ionic radius (R_Li_ = 0.56 Å) for Li ions. Moreover, both the molecular weight (MW, g/mol) and molar volume (V_M_, cm^3^/mol) follow the ρ trend, where they decreased from 102.949 to 77.005 g/mol and from 27.719 to 24.140, for Mw and V_M_, respectively. 

**Figure 3:**
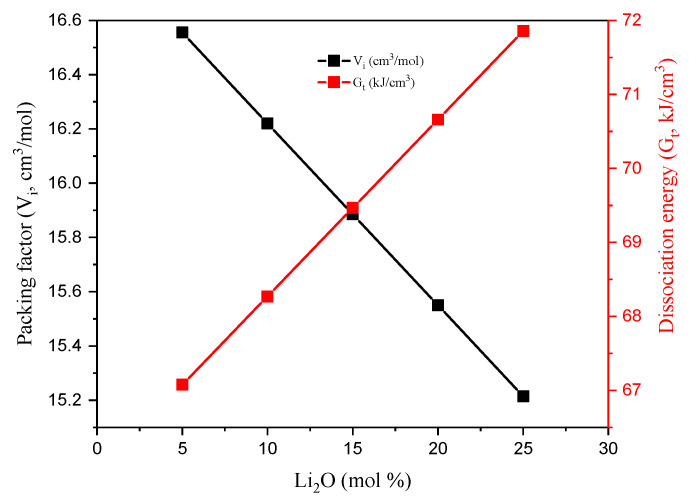
The investigated glasses’ dissociation energy and packing factor versus the Li_2_O concentration.

In order to compute the elastic moduli (EM), Young (Y), shear (K), bulk (B), and longitudinal (L), the Makishima–Mackenzie (M–M) model assumptions were applied. Thus, both the investigated TABLi glasses’ dissociation energy (G_t_) and backing factor (V_i_) were calculated. The G_t_ values were increased by replacing the TeO_2_ with Li_2_O compounds. This is attributed to the heat of formation (enthalpy, ∆H_f_) of constituting compounds, where it is −561.2 kJ/mol for Li_2_O and −270.3 kJ/mol for TeO_2_. On the other hand, the glasses’ packing factor (V_i_, cm^3^/mol) was computed for the TABLi glasses with the help of the values of the ionic radius of Te, B, As, Li, and O. Figure 3 displays a reverse relationship between the V_i_ and Li_2_O concentration. The Vi values decreased from 16.55 to 15.215 cm^3^/mol, increasing TeO_2_ substitution by Li_2_O. This can be attributed to the replacement of Te ions with a higher ionic radius (R_Te_ = 2.22 Å) by Li ions with a smaller Pauli ionic radius (R_Li_ = 0.56 Å). 

The TABLi glasses’ packing density (V_t_) was reported based on the predicted V_i_ values. V_t_’s calculated values were 0.567 to 0.630, raising the ratio of Li ions in the glass network.

**Figure 4:**
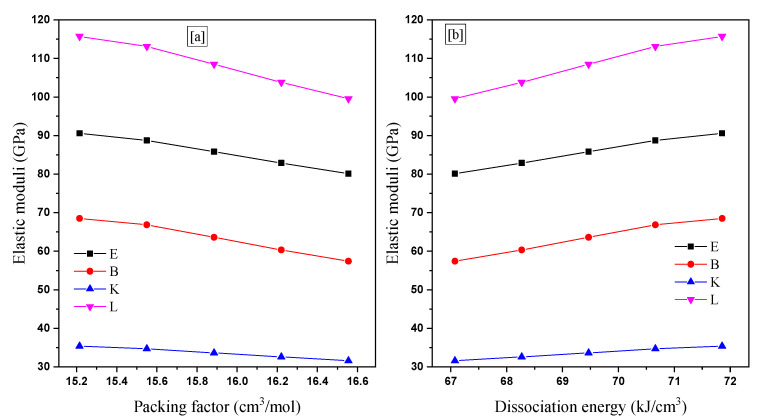
The elastic moduli (Y, B, K, and L) versus (**a**) packing factor and (**b**) dissociation energy.

The elastic moduli (Y, K, B, and L) were reported for the investigated samples. Clearly, Y’s modulus decreased by increasing the V_i_ values. But it is enhanced by increasing the G_t_ of the TABLi glasses, as shown in Figure 4. This is due to the substitution of weak Ti-O bonds by strong Li-O bonds. Additionally, Y’s values enhanced from 80.120 to 90.579 GPa by raising the Li_2_O between 5 and 25 mol%, respectively. The other moduli K, B, and L’s values follow the same trend as Y when the Li_2_O increases between 5 and 25 mol%.

**Figure 5:**
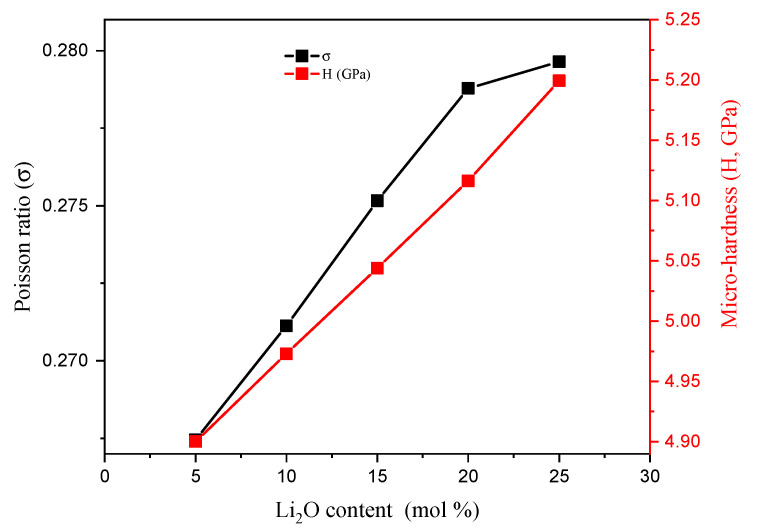
Variation in the Poisson ratio and microhardness for the fabricated TABLi glasses as a function of the Li_2_O insertion ratio.

The hardness (GPa) is considered an important parameter for shielding materials such as concretes and bricks, but in the case of small-scale materials, such as glass, the term microhardness (H, GPa) is applied. It is used to describe the load which the material can stand over without deformations. The TABLi glasses’ microhardness presented in Figure 5 was enhanced from 4.900 to 5.199 GPa by increasing the Li_2_O in the content. This can contribute to the increase in compactness of the material and decrease the r (B-B), r (Li-Li), and r (Te-Te) by increasing the Li ions in the glass network (Table 2). The Poisson ratio (σ) describes the expansion ratio of the investigated TABLi glasses in the direction vertical to the loader direction. Figure 5 showed that the σ values also increase from 0.267 to 0.280 with the replacement of TeO_2_ with Li_2_O content.

**Figure 6:**
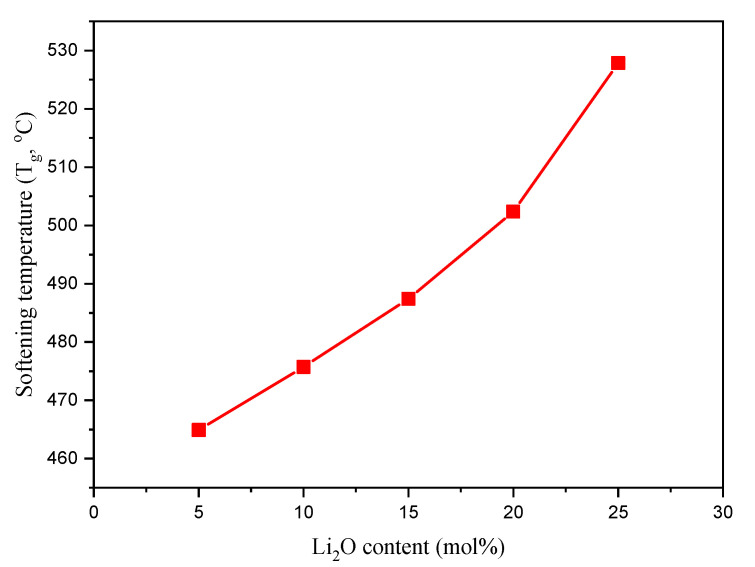
Variation in the softening temperature (T_g_) versus the Ti_2_O insertion ratio.

Softening temperature (T_g_, °C) was reported for the TABLi glasses based on the EM predicted previously (see Figure 6). It is clear that the T_g_’s values increased from 464.9 to 525.8 °C. The increase detected in T_g_ values is due to the replacement of TeO_2_ with a lower melting point (MP) by a higher (i.e., Li_2_O), where the MP_TeO2_ = 733 °C and MP_Li2O_ = 1570 °C.

The fractal bond conductivity (d) for the investigated glass samples decreased from 2.20 to 2.06. This means that the d values are close to two. Thus, the investigated glasses possess a two-dimensional layer structure network.

### 3.2. Shielding Properties

**Figure 7:**
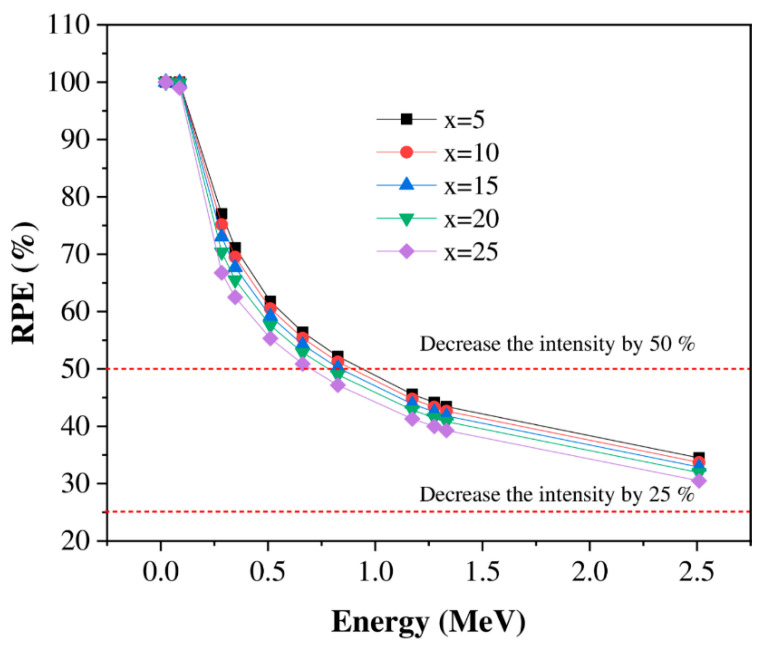
Variation in the investigated glasses’ radiation protection efficiency (RPE) versus the photon energy.

**Figure 8:**
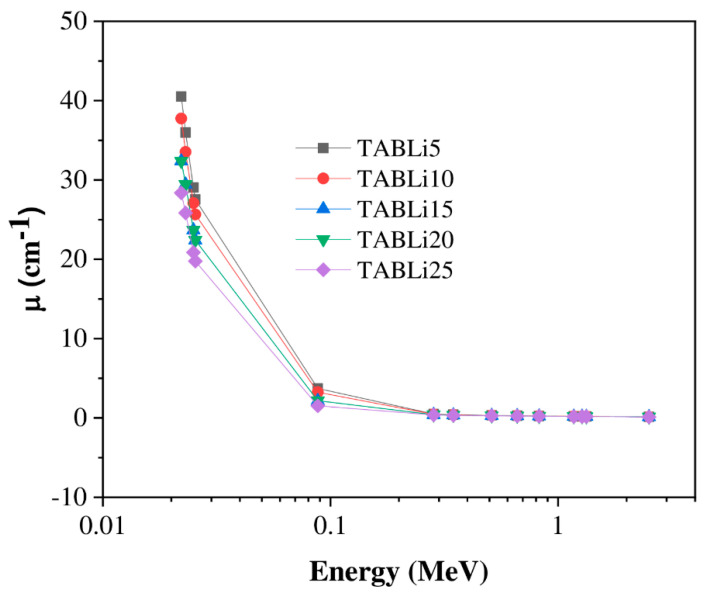
The studied glasses’ linear attenuation coefficient vs the energy.

**Figure 9:**
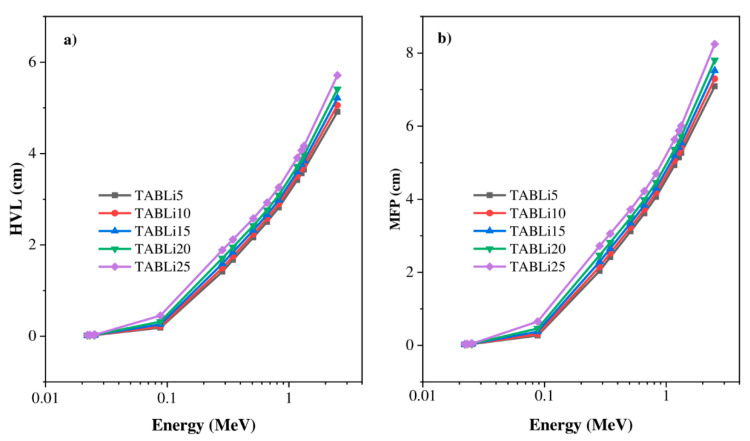
The dependence of the (**a**) half-value layer and (**b**) mean free path on the incoming gamma photons.

**Figure 10:**
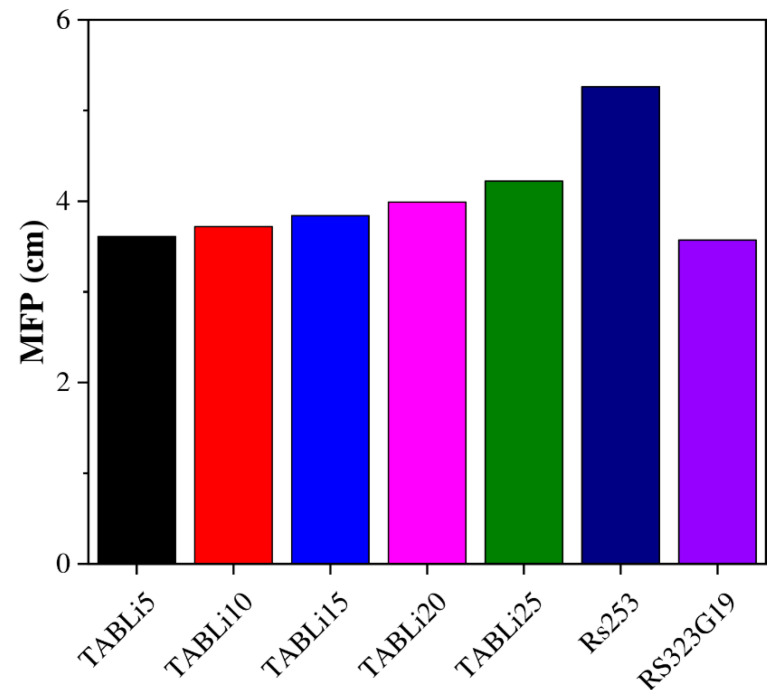
Comparison between the MFP of the studied glasses with some known commercial glasses (RS253 and RS323-G19).

**Figure 11:**
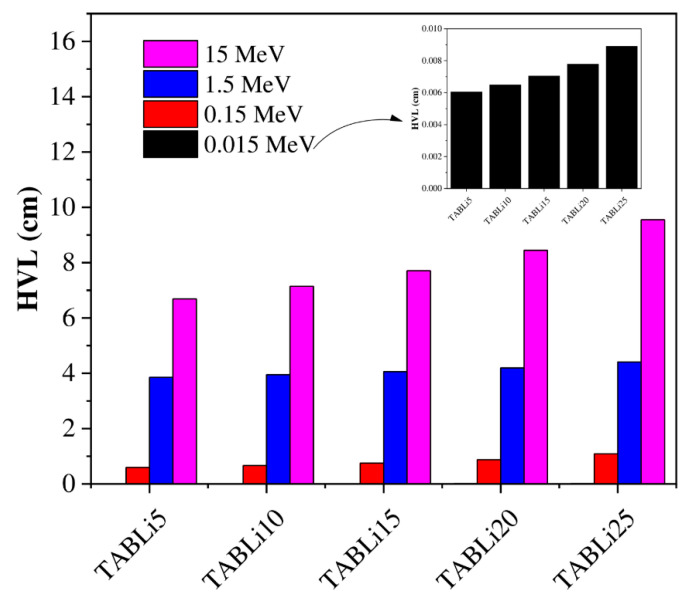
Variation of the investigated glasses’ half-value layer versus LiO_2_ concentration.

**Figure 12:**
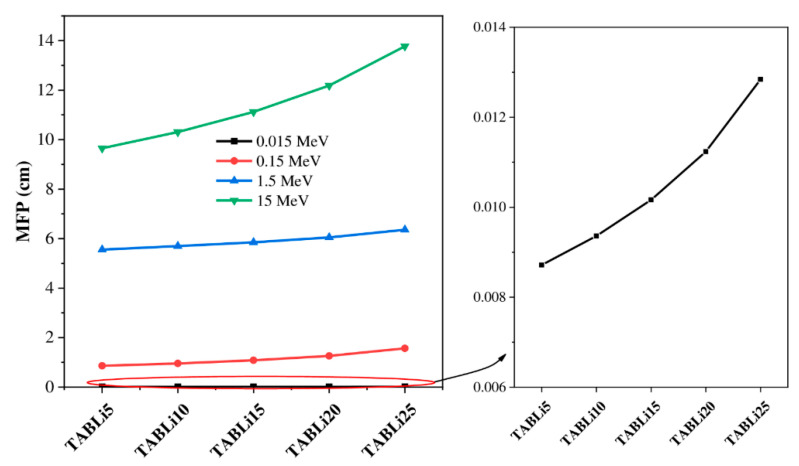
Variation of the investigated glasses’ mean free path versus LiO_2_ concentration.

The glass understudy’s effectiveness in resisting gamma quanta depends on the efficiency of the glass material in absorbing and attenuating incident gamma radiation. Therefore, essential shielding parameters such as radiation protection efficiency (RPE), linear attenuation (LAC), and mean free path (MFP) need to be studied. Awareness of these factors’ performances makes it reasonable to assess the protection efficiency of and the suitable applications for utilizing the glasses to resist radiation. Figure 7, Figure 8, Figure 9, Figure 10, Figure 11 and Figure 12 illustrate the data of simulated radiation shielding parameters computed via the MCNP-5 simulation code. It can be perceived in Figure 7 that the RPE results are influenced by the applied gamma photons’ energy. The increment of the applied gamma energy leads to the decrement of the RPE values for the examined glasses. The following inferences can be interpreted from the examination of the reduction in the gamma radiation intensity with the variation of gamma energies. At low energies (0.0221–0.088 MeV), the investigated glasses appear to be effective at opposing incoming gamma radiation, with RPE values of approximately 100% for all investigated glasses. At the same time, the RPE values diminish with an increase in the gamma radiation intensity from 0.284 to 2.51 MeV based on the Li_2_O concentration in the selected samples. The RPE values of the glasses with a high concentration of Li_2_O (25% mol) have not exceeded 31% in the case of high gamma-quanta energy of 2.51 MeV. Thus, the obtained data for all investigated glasses illustrate that the glasses doped with 5% of Li_2_O content are the preferable glasses that can be applied in the different radiation shielding implementations.

Among the essential parameters of shielding properties is the linear attenuation coefficient (µ), which is used to display the ability of glass material to resist and absorb gamma quanta. Here in the present investigation, the µ is varied between low and high values depending on two parameters: the intensity of gamma-quanta energy and the concentration of dopant (Li_2_O) in the studied glass material. The simulated µ values are deduced from the interaction of the gamma-quanta intensity (I) with the glass material at the known thickness (x), and represented in the following formula: (µ = $\frac{1}{x}\ln\frac{I}{I_{0}}$). The data of µ depends on the interaction type of gamma quanta, and are explained as follows and plotted in Figure 8: the first interaction is a photoelectric effect (PE) which is achieved in the low gamma-quanta energy range (0.0221–0.088 MeV), and the µ data have appeared with the maximum values. 

Successively, the increment of gamma-quanta energy above 0.1 MeV leads to a drop in the µ data as a result of the new interaction, namely Compton scattering (CS). Compton scattering is preponderant, and the inverse relation between CS cross-section and quanta energy was detected where σ_CS_ α E^−1^ [35].

The µ data are manifested with the maximum values at the low applied gamma-quanta energy (0.0221 MeV). It was reduced from 40.5 to 28.3 cm^−1^ for 5% mol and 25% mol of Li_2_O content in the investigated glasses. In contrast, the µ data are observed with the minimum values at the highest applied gamma-quanta energy, 2.51 MeV, where it varies in decrement from 0.14 to 0.12 cm^−1^ for 5% mol and 25% mol of Li_2_O content, respectively. 

Furthermore, the µ data impacted the insertion of Li_2_O concentration in the studied glasses. At stationary gamma-quanta energy, the µ data are diminished with the addition of Li_2_O content from 5 mol% to 25 mol% due to the molecular weight decrease from 102.95 to 77.01 g/mol for 5 mol% and 25 mol% of Li_2_O content. Therefore, the effective atomic number (Z_eff_) decreases. The maximum data of µ lessened in-between 40.5 and 0.14 cm^−1^ for the studied glasses with 5 mol% content of Li_2_O. The minimum data varied in decrement 8.89–0.03 cm^−1^ and established at the examined glasses with 25 mol% content of Li_2_O. Finally, the replacement of TeO_2_ content with Li_2_O content procures the decrement of µ data due to the direct proportionality between the cross-section of CS and the effective atomic number where σ_CS_ α Z_eff_.

Agreement was detected between the obtained µ data with the kinds in the literature concerned with various types of tellurite glasses [36,37,38].

The other parameter is the mass attenuation coefficient (µ/ρ), which was computed via the MCNP-5 code based on the density of the investigated glasses, and compared with the theoretical data of µ/ρ, which was detected by Phy-X/PSD. The difference Δ (%) between the simulated and theoretical data was estimated by the next formula [39] and presented in Table 3:

(10) Δ (%)=[(μρ)MCNP−(μρ)Phy−X/PSD](μρ)MCNP×100 

The difference Δ (%) observed did not exceed 10% between all investigated TABLi glasses.

The HVL and the MFP are the radiation protection factors used to minimize the applied gamma-quanta energy to a half and display the distance between successive interactions. Contrarily, the µ data of simulated HVL are observed to rise with the applied gamma-quanta energy and the addition of Li_2_O concentration in the TABLi glasses, as shown in Figure 9. The following formulas are applied to estimate the HVL and MFP:

(11) $$\mathrm{HVL}\;\left(\mathrm{cm}\right)=\frac{\ln2}{\mu }\;\&\;\mathrm{MFP}\;\left(\mathrm{cm}\right)=\frac{1}{\mu }\;$$

The results presented in Figure 9 illustrate that an increment in the concentration of Li_2_O in the glasses’ structure and the creation of supplementary absorption bands assist in the reality that for dopant Li_2_O contents of 5–25 mol% in the investigated glasses, the HVL and MFP diminish by 1.5 times. The difference revealed that the insertion of Li_2_O could significantly decrease the thickness of the investigated glasses, without losing efficiency and minimizing the costs of production. The HVL data that reached the maximum values at the high gamma-quanta energy of 2.51 MeV ranged from 4.9 to 5.7 cm for TABLi5 and TABLi25, respectively, as well as the MFP data, which varied in an increase of 7.1 and 8.2 for TABLi5 and TABLi25, respectively. Furthermore, the investigated glasses’ low HVL and MFP data are detected at the low applied gamma-quanta energy (0.0221 MeV). Moreover, the glasses’ understudy with the lower content of Li_2_O (5 mol%) is considered better than glasses with a high content of Li_2_O. Consequently, it can be used in radiation protection applications. Based on the MFP data, the present glasses are compared with the commercial glasses RS253 and RS323-G19 [40] and plotted in Figure 10. The comparison displayed that the MFP values of studied glasses are lower than RS253 and comparable with the synthetic glasses RS323-G19. This means the examined glasses are suitable for application in the radiation protection fields, especially the glasses with 5% mol of Li_2_O content (TABLi5) since the mean voyaged distance between two photo interactions is small. 

Figure 11 and Figure 12 depict the injection of Li_2_O concentration in the tellurite glasses that impacted the HVL and MFP data. The Phy-x/PD computer program was employed to theoretically estimate the HVL and MFP data of the synthetic glasses. It is plain in Figure 11 and Figure 12 that the TABLi5 glasses have the lowest data of HVL and MFP while the TABLi25 glasses have the highest data at all chosen gamma-quanta energies (0.015, 0.15, 1.5, and 15 MeV), and this agrees with their simulated data. 

**Figure 13:**
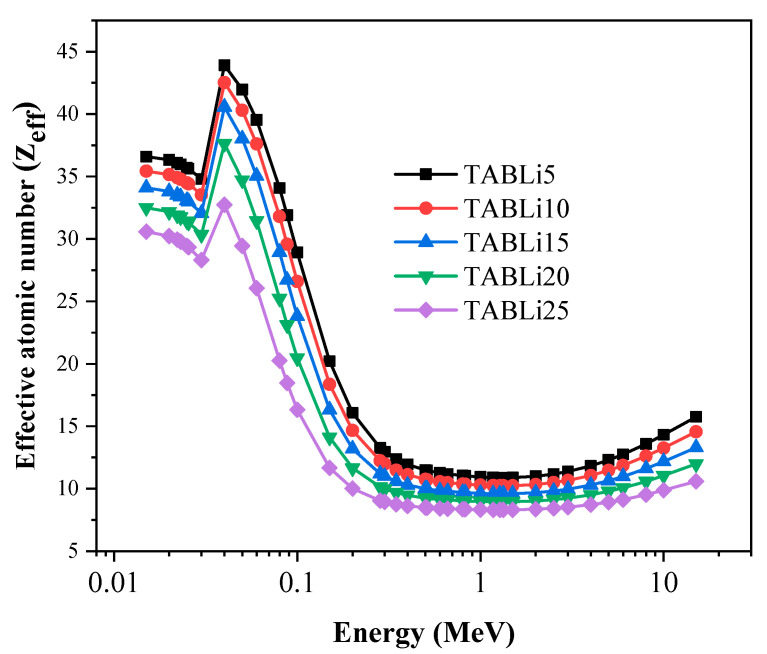
Variation of the studied glasses’ effective atomic number (Z_eff_) versus the gamma photon energy.

**Figure 14:**
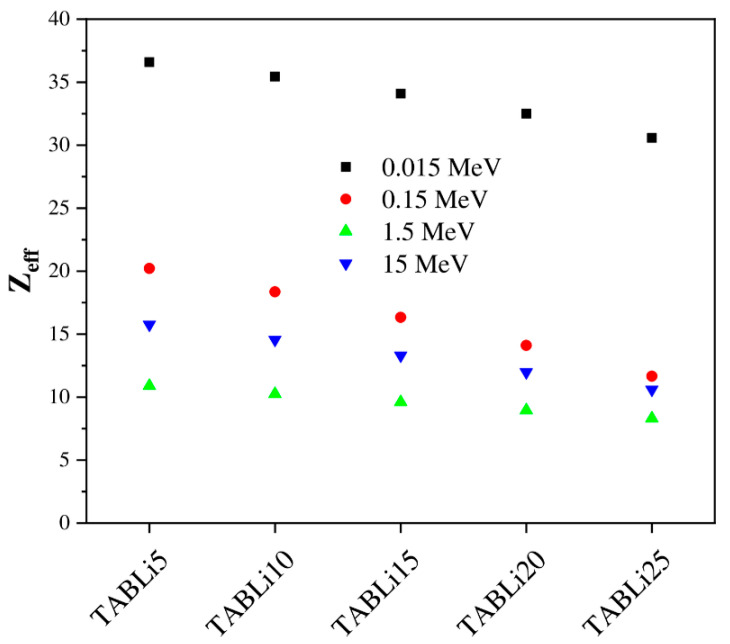
The dependence of the studied glasses’ effective atomic number (Z_eff_) on the LiO_2_ concentration.

**Figure 15:**
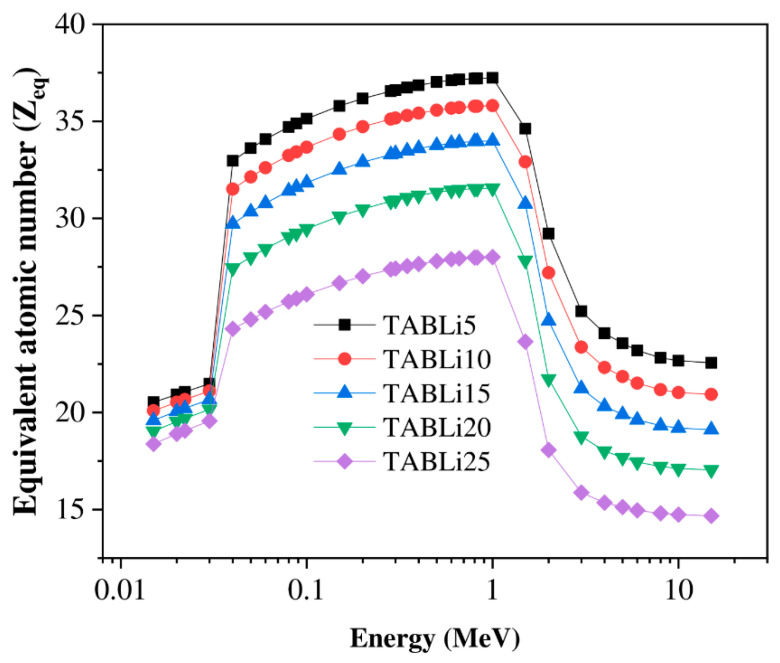
The equivalent atomic number as a function of the energy.

**Figure 16:**
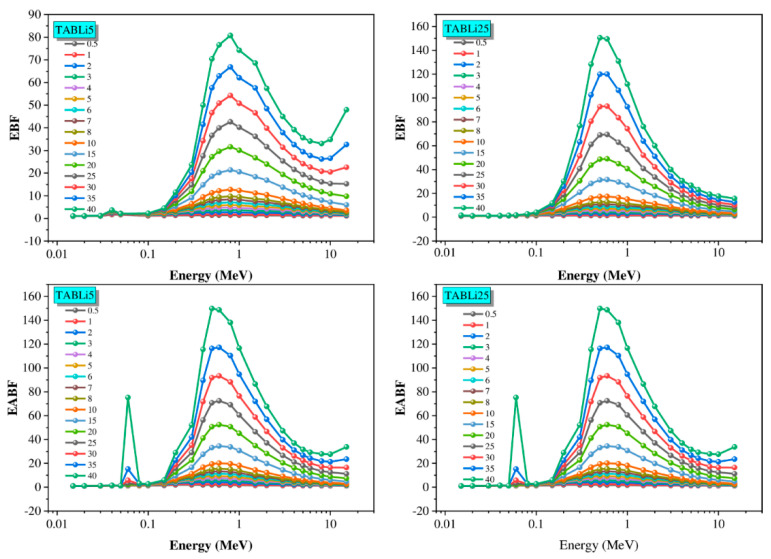
The buildup factorsversus the energy.

**Figure 17:**
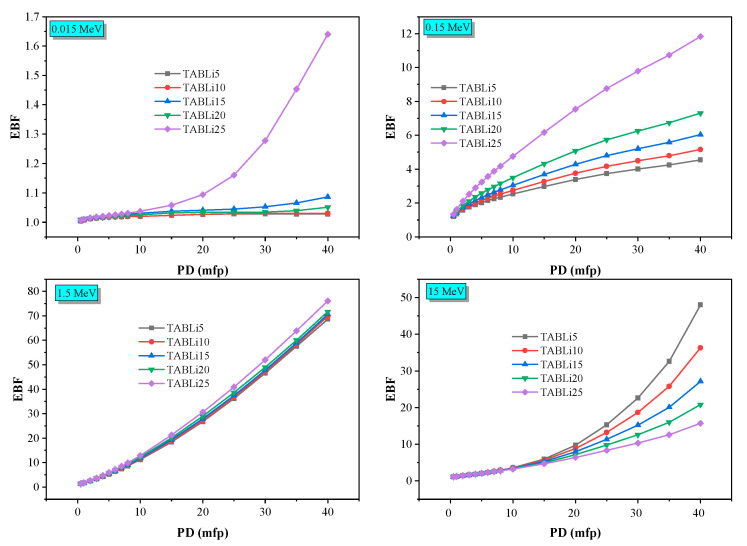
The exposure buildup factor (EBF) dependent on the penetration depth values (PD) at some fixed energies.

**Figure 18:**
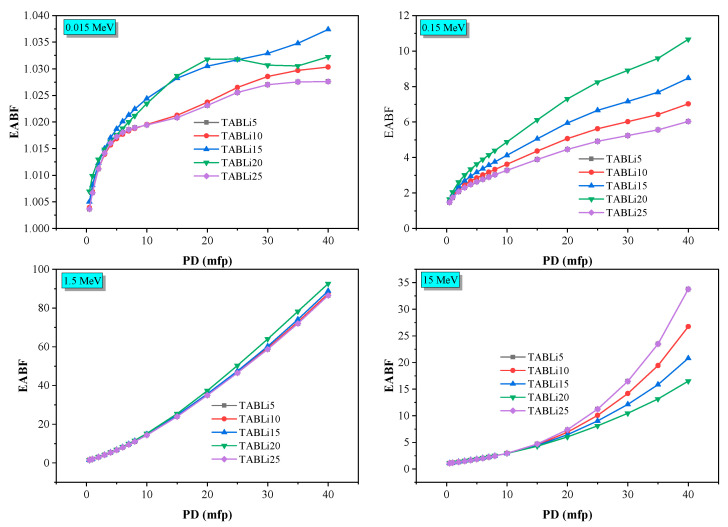
The EABF dependent on the penetration depth values (PD) at some fixed energies.

Furthermore, the Phy-x/PD was employed to compute radiation protection items, including glasses’ effective atomic number (Z_eff_), equivalent atomic number (Z_eq_), as well as the accumulation factors, those being exposure buildup factors (EBF) and energy absorption buildup factor (EABF). Figure 13, Figure 14, Figure 15, Figure 16, Figure 17 and Figure 18 depicted the acquired data, which were then addressed in the lines below.

Figure 13 reveals the effective atomic number (Z_eff_) data that are designated to investigate the capacity of the synthetic glasses for serving in the implementations of gamma shielding. The data of Z_eff_ are changed with the gamma-quanta energy (0.015–15 MeV) and the Li_2_O concentration (5–25 mol%) in the examined TABLi glasses. The Z_eff_ data are influenced by the gamma quanta interaction with the glass material. For low gamma-quanta energy range (0.015–0.1 MeV), the photoelectric effect (PE) interactions are dominant, and the maximum Z_eff_ data seem to be where Z^4^ varied. After that, the Z_eff_ data diminished when the gamma-quanta energy increased. However, unpredicted peaks are observed at gamma-quanta energy 0.0318 MeV [41].

Then, the Compton scattering interactions are begun to possess gamma-quanta energy in the range of above 0.1 MeV, and the data of Z_eff_ are observed gradually in decrement where it altered with the atomic number (Z). The increase in gamma-quanta energy at high values leads to pair production, which varied with Z^2^ [42]. Additionally, the Z_eff_ data reduced, as illustrated in Figure 14.

Figure 15 offers the equivalent atomic number (Z_eq_) at various gamma photon energy. It is estimated according to the values of µ/ρ in addition to the atomic numbers of elements (Z1 and Z2) related to the ratios R1 and R2 as well as the ratio for the examined glasses at stationary gamma-quanta energy. Thus, Z_eq_ is estimated by the following formula (Z_eq_ = $\frac{Z1\;(\log R2-\log R)+Z2\;\left(\mathrm{logR}-\mathrm{logR}1\right)}{\mathrm{logR}2-\mathrm{logR}1}$).

It can be noticed that the Z_eq_ data increased with the increment of gamma-quanta energy up to 1 MeV. The maximum values of Z_eq_ are founded in the CS region (energy > 1 MeV). The maximum data of Z_eq_ are 37.23 and 28.01 for TABLi5 and TABLi25, respectively, while the minimum data are 22.56 and 14.68 for TABLi5 and TABLi25, respectively.

The total flux of gamma quanta in the studied glass material can be determined by utilizing two main buildup and accumulation factors: the EBF and the EABF. The alteration of EBF and EABF data with the gamma-quanta energy for the TABLi5 and TABLi25 glasses is plotted in Figure 16. The EBF and EABF data are computed according to the Z_eq_ values and the approximation of G-P fitting [43,44]. For instance, the Z_eq_ data and the five factors of GP fitting are presented in Table 4 for TABLi5 and TABLi25 glasses, respectively.

Figure 16 exhibits the variety of EBF and EABF data with the gamma-quanta energy up to 15 MeV. The EBF and EABF data seem to have their minimum values in the low gamma-quanta energy zone. This is due to the gamma photons passing through the studied glass material linked with the PE phenomena. In this region, the sharp peaks of both factors are reported around the quanta energy (0.0318 MeV). The increment of quanta energy leads to the increase in the accumulation of gamma photons inside the material, where the CS process is significant. The drop photons interacted with and penetrated the studied glass thickness while the rest of the photons are scattered to induce multiple interactions. In the CS region, the EBF and EABF data reach their maximum values. After that, it can be seen that the both factors began to decrease when the gamma-quanta energy had high values thanks to the third interaction as represented in the PP. (Mahmoud et al., 2020; YS. Rammah et al., 2020). 

Furthermore, Figure 17 and Figure 18 show that the EBF and EABF data impacted the penetration depth, which changes from 0.5 to 40 mfp at four specified gamma-quanta energies of 0.015, 0.15, 1.5, and 15 MeV, as well as the chemical composition of TABLi glasses. 

Figure 17 and Figure 18 manifested the exchange of EBF and EABF data with the penetration depth (PD) at identified gamma-quanta energy (0.015, 0.15, 1.5, and 15 MeV) prominently. The photon accumulation inside the glass material is associated with the distance that photons will travel as well as the time spent inside the investigated material. The low EBF and EABF data are identified at the short traveling distance (0.5 mfp), while the highest data was achieved at the long traveling distance (40 mfp). Moreover, the alteration of Li_2_O within the examined glass material was due to the elevation of EBF and EABF.

**Figure 19:**
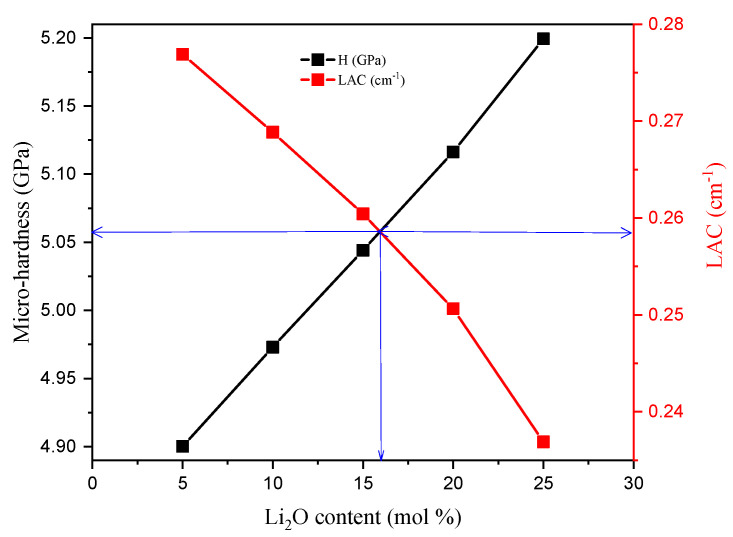
Relationship between the microhardness and the linear attenuation coefficient (LAC) for the various investigated samples.

Figure 19 showed the variation of the LAC and the microhardness (H) versus the Li_2_O concentration. The LAC of the investigated samples diminished while the H values enhanced by increasing the Li_2_O substitution ratio. The glass sample with Li_2_O content of 5 mol% (TABLi5) has the highest LAC (LAC = 0.276 cm^−1^) at energy 0.662 MeV, but the microhardness of the mentioned sample is relatively low (H = 4.900 GPa). In contrast, the sample with Li_2_O content of 25 mol% (TABLi25) has the lowest LAC value (LAC = 0.236 cm^−1^), but it has the highest H value (H = 5.199 GPa). Thus, the correlation showed in Figure 19 is used to predict which sample has both LAC and H suitable values. According to this relation, the sample with a suitable LAC and H contains around 16 mol% of Li_2_O concentration. The microhardness of this glass (i.e., containing 16 mol% of Li_2_O) is around 4.055 GPa, and the LAC is about 2.585 cm^−1^.

## 4. Conclusions

The microhardness and softening temperature, elastic moduli and Poisson ratio were calculated based on the M–M model. The microhardness and Poisson ratio were enhanced by the replacement of the TeO_2_ by the Li_2_O. The H values increased from 4.90 to 5.20 GPa, and σ values rose from 0.267 to 0.280, raising the Li_2_O concentration between 5 and 25 mol%, respectively. The elastic Young, shear, longitudinal, and bulk modules were enhanced by increasing the Li ions in the glass network. Also, the shielding characteristics showed that the LAC was diminished with the replacement of Te by Li ions in the glass network. The LAC decreased from 30–90 to 24.40 cm^−1^ at 0.015 MeV when the Li_2_O concentration increased between 5 and 25 mol%, respectively. The HVLand MFP values increased by raising the Li_2_O concentration in the glass network. Additionally, the accumulation of photons in air EBF and inside the glass layers (EABF) was increased by increasing the Li_2_O concentration.

## Figures and Tables

**Table 1:** The chemical composition of the investigated glass samples.

	Composition (wt%)				Density (g/cm^3^)	MW (g/mol)	Vm (cm^3^/mol)
	As_2_O_3_	B_2_O_3_	Li_2_O	TeO_2_			
TABLi5	19.217	40.574	1.451	38.757	3.71	102.95	27.72
TABLi10	20.510	43.303	3.098	33.090	3.61	96.46	26.73
TABLi15	21.988	46.424	4.982	26.607	3.50	89.98	25.72
TABLi20	23.696	50.030	7.158	19.116	3.37	83.49	24.77
TABLi25	25.692	54.244	9.701	10.363	3.19	77.01	24.14

**Table 2:** Some mechanical properties for the glasses under study.

	TABLi5	TABLi10	TABLi15	TABLi20	TABLi25
V_t_	0.60	0.61	0.62	0.63	0.63
V_l_ (m/s)	5177.61	5362.85	5568.19	5792.38	6022.41
V_s_ (m/s)	2917.22	3005.14	3101.19	3207.99	3330.88
Fractal bond conductivity	2.20	2.16	2.12	2.08	2.07
r(B-B), Å	2.56	2.53	2.49	2.46	2.44
r(Li-Li), Å	2.82	2.77	2.71	2.65	2.61
r(Te-Te) Å	2.79	2.77	2.73	2.70	2.67

**Table 3:** The mass attenuation coefficient of the chosen glasses.

Energy (MeV)	Mass Attenuation Coefficient (cm^2^/g)														
	TABLi5			TABLi10			TABLi15			TABLi20			TABLi25		
	MCNP-5	Phy-X/PSD	∆ (%)	MCNP-5	Phy-X/PSD	∆ (%)	MCNP-5	Phy-X/PSD	∆ (%)	MCNP-5	Phy-X/PSD	∆ (%)	MCNP-5	Phy-X/PSD	∆ (%)
0.015		30.89			29.60			28.12			26.41			24.41	
0.022	10.91	10.92	−0.07	10.46	10.46	−0.05	9.95	9.95	−0.02	9.61	9.35	2.67	8.89	8.65	2.71
0.023	9.69	9.69	−0.04	9.29	9.29	−0.01	8.83	8.83	0.03	8.73	8.30	4.94	8.10	7.68	5.18
0.025	7.82	7.84	−0.18	7.50	7.51	−0.15	7.13	7.14	−0.10	7.02	6.71	4.42	6.54	6.21	4.99
0.026	7.41	7.43	−0.23	7.11	7.12	−0.20	6.76	6.77	−0.15	6.65	6.37	4.26	6.19	5.89	4.88
0.030		4.82			4.62			4.39			4.12			3.82	
0.050		4.19			3.71			3.17			2.54			1.80	
0.080		1.25			1.12			0.96			0.79			0.59	
0.088	0.95	0.98	−4.20	0.90	0.88	1.37	0.76	0.77	−0.58	0.64	0.64	1.26	0.48	0.48	0.45
0.100		0.73			0.66			0.58			0.48			0.37	
0.150		0.31			0.29			0.26			0.23			0.20	
0.284	0.13	0.13	−0.33	0.13	0.13	−0.32	0.12	0.13	−0.30	0.12	0.12	−0.28	0.12	0.12	−0.25
0.300		0.13			0.12			0.12			0.12			0.11	
0.347	0.11	0.11	−0.18	0.11	0.11	−0.17	0.11	0.11	−0.16	0.11	0.11	−0.15	0.10	0.10	−0.13
0.500		0.09			0.09			0.09			0.09			0.09	
0.511	0.09	0.09	−0.18	0.09	0.09	−0.17	0.09	0.09	−0.16	0.08	0.08	−0.14	0.08	0.08	−0.12
0.662	0.07	0.07	−0.22	0.07	0.07	−0.21	0.07	0.07	−0.19	0.07	0.07	−0.18	0.07	0.07	−0.16
0.800		0.07			0.07			0.07			0.07			0.07	
0.826	0.07	0.07	−0.19	0.07	0.07	−0.18	0.07	0.07	−0.17	0.07	0.07	−0.16	0.07	0.07	−0.14
1		0.06			0.06			0.06			0.06			0.06	
1.173	0.05	0.06	−0.90	0.05	0.06	−0.83	0.06	0.06	−0.75	0.06	0.06	−0.66	0.06	0.06	−0.55
1.275	0.05	0.05	−0.76	0.05	0.05	−0.70	0.05	0.05	−0.63	0.05	0.05	−0.55	0.05	0.05	−0.46
1.333	0.05	0.05	−0.72	0.05	0.05	−0.66	0.05	0.05	−0.59	0.05	0.05	−0.52	0.05	0.05	−0.43
1.5		0.05			0.05			0.05			0.05			0.05	
2.506	0.04	0.04	−0.45	0.04	0.04	−0.43	0.04	0.04	−0.40	0.04	0.04	−0.37	0.04	0.04	−0.34
3		0.04			0.04			0.04			0.04			0.04	
5		0.03			0.03			0.03			0.03			0.03	
8		0.03			0.03			0.03			0.03			0.02	
10		0.03			0.03			0.03			0.02			0.02	
15		0.03			0.03			0.03			0.02			0.02	

**Table 4:** The GP fitting parameters for the TALBi5 sample at various gamma energies.

E (MeV)	Z_eq_	EBF					EABF				
		a	b	c	d	Xk	a	b	c	d	Xk
0.015	20.53	−0.04	1.01	0.69	0.15	6.89	−0.02	1.01	0.68	0.14	9.31
0.02	20.93	0.26	1.02	0.39	−0.18	10.98	0.26	1.02	0.35	−0.20	12.47
0.03	21.49	0.22	1.05	0.37	−0.17	16.39	0.24	1.05	0.35	−0.16	14.46
0.04	32.96	0.19	2.04	0.33	−0.07	17.54	0.17	1.19	0.37	−0.20	24.68
0.05	33.61	0.02	1.88	0.23	−0.06	12.52	0.08	1.20	0.23	−0.04	11.06
0.06	34.08	0.61	1.67	0.21	−0.14	15.27	0.49	1.21	0.20	−0.17	14.70
0.08	34.71	0.47	1.42	0.25	−0.18	14.18	0.39	1.27	0.24	−0.17	14.22
0.1	35.13	0.23	1.22	0.40	−0.13	13.82	0.23	1.28	0.39	−0.13	16.50
0.15	35.79	0.14	1.36	0.57	−0.08	14.23	0.25	1.74	0.39	−0.15	13.85
0.2	36.17	0.12	1.56	0.64	−0.07	14.02	0.24	2.41	0.45	−0.16	13.80
0.3	36.61	0.05	1.69	0.84	−0.03	13.76	0.11	2.59	0.69	−0.08	13.59
0.4	36.85	0.02	1.78	0.98	−0.03	13.22	0.07	2.72	0.84	−0.07	13.30
0.5	37.02	0.00	1.82	1.05	−0.02	12.83	0.04	2.67	0.94	−0.05	13.10
0.6	37.11	−0.01	1.83	1.09	−0.02	12.13	0.02	2.59	0.99	−0.04	12.75
0.8	37.20	−0.02	1.82	1.12	−0.01	11.73	0.01	2.43	1.05	−0.03	12.06
1	37.23	−0.02	1.79	1.13	−0.01	11.49	0.00	2.29	1.07	−0.02	11.46
1.5	34.62	−0.03	1.68	1.17	0.01	12.99	−0.02	1.95	1.12	−0.01	10.71
2	29.21	−0.02	1.69	1.12	0.00	8.97	−0.02	1.84	1.11	−0.01	9.56
3	25.22	−0.01	1.63	1.06	−0.01	12.14	0.00	1.68	1.04	−0.02	12.39
4	24.08	0.01	1.56	1.02	−0.02	12.67	0.01	1.57	0.99	−0.03	13.43
5	23.57	0.01	1.49	1.00	−0.02	13.15	0.02	1.48	0.97	−0.04	14.30
6	23.20	0.02	1.45	0.97	−0.03	13.29	0.03	1.41	0.96	−0.04	13.75
8	22.82	0.03	1.36	0.96	−0.04	13.59	0.03	1.31	0.95	−0.04	13.19
10	22.67	0.04	1.31	0.94	−0.05	13.78	0.04	1.26	0.93	−0.05	14.08
15	22.56	0.05	1.21	0.93	−0.06	14.12	0.04	1.16	0.96	−0.04	14.54

## Data Availability

The data presented in this study are available on request from the corresponding author.
